# Next-Generation Strategies for Controlling Foodborne Pathogens: Precision Antimicrobials, Biofilm Disruption, and Emerging Molecular Interventions

**DOI:** 10.3390/foods15020194

**Published:** 2026-01-06

**Authors:** Ayman Elbehiry, Ahmed I. Alajaji

**Affiliations:** 1 Department of Public Health, College of Applied Medical Sciences, Qassim University, P.O. Box 6666, Buraydah 51452, Saudi Arabia; ar.elbehiry@qu.edu.sa; 2 Department of Veterinary Preventive Medicine, College of Veterinary Medicine, Qassim University, Buraydah 51452, Saudi Arabia

**Keywords:** precision food safety, foodborne pathogens, biofilm control, antibiotic resistance, bacteriophages, nanotechnology, surface engineering, multi-hurdle preservation

## Abstract

Foodborne diseases remain a major global challenge because pathogenic microorganisms persist in food systems, often protected by biofilms and increasing resistance to conventional chemical preservatives and sanitizers. Control strategies that were effective in the past are becoming less reliable in complex processing environments, creating a need for more precise and adaptable food-safety approaches. This review examines emerging technologies that shift food safety from broad, reactive control toward targeted, data-driven intervention. Biological tools, including bacteriophages, phage-derived enzymes, bacteriocins, quorum-sensing inhibitors, and gene-guided antimicrobial systems, are discussed for their capacity to selectively control specific pathogens while limiting unintended effects on beneficial microbiota. The review also addresses nano-enabled strategies that improve antimicrobial stability, delivery, and performance, along with plant-derived and microbial bioactive compounds that support clean-label and sustainable preservation. In parallel, advances in anti-biofilm surface engineering, such as nano-textured, contact-active, and responsive materials, are examined as preventive measures to reduce microbial attachment and persistence on food-contact surfaces. Beyond individual interventions, this review emphasizes integration within coordinated multi-hurdle systems supported by real-time monitoring and predictive analytics. Emerging digital frameworks, including digital twins of food-processing lines, are highlighted as tools to link detection, risk prediction, and targeted control. Finally, remaining knowledge gaps, regulatory challenges, and research priorities are identified, highlighting the need for realistic testing, long-term safety evaluation, standardized validation, and collaborative efforts to translate precision food-safety technologies into dependable real-world applications.

## 1. Introduction

Foodborne disease remains a major global public health concern. Current estimates indicate that unsafe food causes approximately 600 million illnesses and 420,000 deaths each year, with children under five accounting for nearly 30% of these fatalities [1,2]. At the same time, conventional control measures, including chemical sanitizers, antibiotics, and thermal or physicochemical treatments, are becoming less effective as antimicrobial resistance (AMR) continues to increase across human, animal, and food-associated environments. Global analyses report consistent upward trends in resistance among major pathogenic bacteria, undermining both clinical treatment and routine infection-control practices [3,4]. These resistance trends are reported across regions and are associated with higher treatment failure rates and increased health and economic burdens, underscoring the need for alternative control strategies that can complement or, in some contexts, replace traditional antibiotics and chemical sanitizers to which foodborne pathogens have developed resistance in food production systems [5].

The capacity of foodborne bacteria to form biofilms and adopt stress-adapted physiological states further complicates control efforts. Many foodborne bacteria, including *Listeria monocytogenes* (*L. monocytogenes*), *Salmonella* spp., and several *Vibrio* and *Staphylococcus* strains, can form biofilms or enter stress-adapted states that reduce susceptibility to disinfectants and promote persistence in food-processing environments [5,6,7]. Biofilms create protected microenvironments that limit the effectiveness of mechanical cleaning and biocides and can act as reservoirs for recurrent contamination [7,8]. In addition, chemical sanitizers may lose activity in the presence of organic matter, and repeated or sublethal exposure can select for stress-adapted populations with reduced sanitizer sensitivity [9,10,11]. These limitations highlight the need for control strategies that remain effective across diverse processing conditions and contamination scenarios.

Rising AMR, persistent biofilms, and growing consumer demand for minimally processed foods have accelerated interest in targeted and sustainable alternatives to broad-spectrum chemical approaches [12,13]. Several emerging technologies now offer more precise and adaptable methods for controlling foodborne pathogens. Biological strategies, particularly bacteriophages and phage-derived enzymes, use natural bacterial predators to achieve highly specific antimicrobial activity and have undergone regulatory evaluation, including Generally Recognized as Safe (GRAS) status for selected applications [13,14].

Nano-enabled antimicrobials represent another active research area. These materials can improve antimicrobial delivery, extend activity, and enhance penetration into biofilms, although concerns related to particle migration, toxicity, and regulatory acceptance currently limit widespread industrial adoption [15,16]. Quorum-sensing inhibitors (QSIs) provide an alternative approach by disrupting bacterial communication systems that regulate biofilm formation and virulence. Because these compounds target behavior rather than viability, they may impose lower selective pressure for resistance development [17,18].

CRISPR-based antimicrobials are emerging as highly specific tools capable of targeting particular bacterial strains or resistance-associated genetic elements, with programmable CRISPR-Cas systems shown to selectively remove targeted bacteria or resistance genes in mixed populations [19]. Despite their promise, practical application in food systems remains constrained by challenges related to delivery, environmental containment, and regulatory approval [20]. In parallel, natural bioactive compounds, including essential oils (EOs), plant polyphenols, and bacteriocins, continue to attract attention because they align with clean-label preferences. Advances in encapsulation and controlled-release technologies have improved their stability and performance in complex food matrices [21,22].

**Figure 1:**
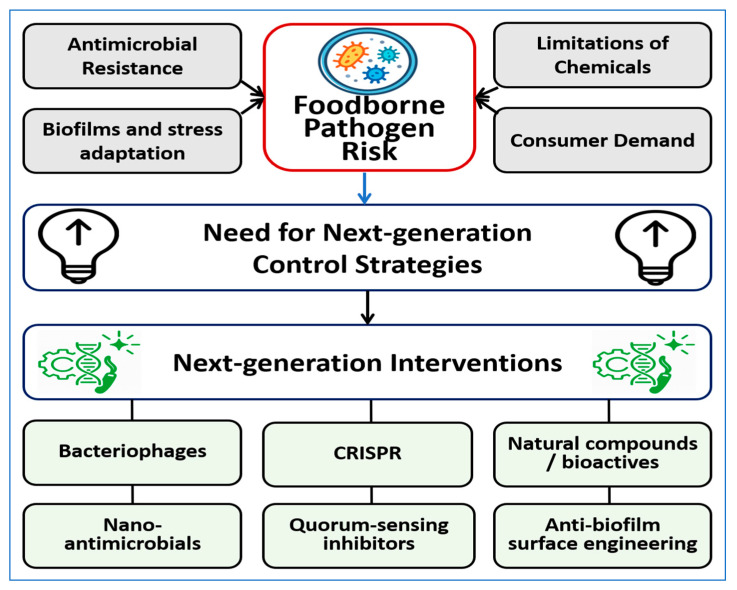
Conceptual overview of the main drivers contributing to foodborne pathogen risk and the rationale for next-generation control strategies. Antimicrobial resistance, biofilm formation and stress adaptation, consumer demand for minimally processed foods, and limitations of conventional chemical controls collectively drive the need for alternative approaches. The figure highlights major categories of emerging interventions examined in this review, including bacteriophages, nano-antimicrobials, quorum-sensing inhibitors, CRISPR-based tools, natural bioactive compounds, and anti-biofilm surface engineering.

Surface engineering strategies are being developed to reduce contamination in processing environments by limiting microbial attachment and biofilm formation, with antimicrobial polymer coatings on steel surfaces shown to substantially reduce bacterial colonization [23]. Approaches such as nano-textured surfaces, superhydrophobic coatings, photocatalytic materials, and enzyme-functionalized coatings aim to prevent pathogen persistence on food-contact surfaces [24,25,26]. The major drivers of foodborne pathogen risk, including antimicrobial resistance, biofilm formation and stress adaptation, consumer demand for minimally processed foods, and limitations of conventional chemical sanitizers, are illustrated in Figure 1. These factors highlight the need for next-generation control strategies and provide context for the intervention categories discussed in this review.

Although many of these technologies show strong performance in laboratory and pilot-scale studies, their adoption in real food systems remains limited. Key challenges include effective delivery in complex matrices, ecological and toxicological concerns, insufficient long-term field data, and regulatory frameworks that are still evolving for next-generation interventions [13,15,27]. Consequently, there is a clear need for comprehensive, evidence-based evaluation of emerging food-safety strategies that accounts for mechanisms of action, real-world performance, regulatory readiness, and integration within multi-hurdle systems [28]. Such a synthesis can help define realistic research priorities and support informed decision-making in both research and industry [13,29].

## 2. Shifting Paradigms in Foodborne Pathogen Control

### 2.1. From Broad-Spectrum to Precision Antimicrobials

Food safety has traditionally relied on broad-spectrum chemical sanitizers and, in limited contexts, antibiotics to reduce microbial contamination [30,31]. While these approaches decrease overall microbial loads, they also affect non-target organisms and can promote the selection of tolerant or resistant populations [8,31]. In contrast, precision antimicrobials are designed to target specific pathogens, genetic traits, or ecological niches within the food chain. These approaches include bacteriophages, phage-derived enzymes, clustered regularly interspaced short palindromic repeats (CRISPR), and tailored nano-formulations [31,32].

By narrowing their spectrum of activity, precision strategies can preserve beneficial microbiota and reduce selective pressure for broad AMR [33]. They also enable interventions to be applied at defined risk points where a particular organism or gene is responsible for contamination [32,34]. This capacity for targeted action improves compatibility with modern food-safety systems that demand both effectiveness and specificity.

### 2.2. From Reactive Decontamination to Proactive Prevention

Traditional sanitation practices are largely reactive, with interventions applied after contamination has occurred. This model often depends on repeated use of intensive chemical treatments [30]. Proactive prevention aims instead to limit contamination before it becomes established. Preventive strategies focus on inhibiting initial surface attachment, disrupting early biofilm development, or interfering with microbial signaling pathways that support persistence [5,35].

Early intervention is critical because mature biofilms display substantial tolerance to disinfectants. Preventing biofilm formation reduces reliance on aggressive sanitation measures and can improve product stability and workplace safety [36]. Effective implementation depends on rapid detection, routine environmental monitoring, and interventions that function reliably under real processing conditions [37,38]. These elements support a more robust and preventive approach to food-safety management.

### 2.3. Integration of Molecular Tools into Food-Safety Systems

Molecular technologies increasingly extend beyond detection into targeted control. Bacteriophages, CRISPR-Cas antimicrobials, and phage-derived lysins can act at the species or gene level, offering high specificity toward defined targets [32]. When paired with rapid detection platforms, these tools enable closer coupling between pathogen identification and intervention. For example, CRISPR-based biosensors provide sequence-specific detection and can be combined with nano-enabled signal amplification to support timely and targeted responses [35,39].

Genomic and other “omics” approaches further improve intervention design by characterizing strain diversity, resistance determinants, and stress-adaptation traits. This information supports selection of appropriate control agents and improves prediction of where interventions are most likely to succeed [8,40]. In parallel, data-driven tools, including predictive models, digital twins, and machine-learning approaches, can integrate sensor data with process information to guide decisions on when and where to apply targeted food-safety controls [41]. Although early studies demonstrate promise, challenges remain related to data standardization, model validation, and pathways for industrial implementation [34,38].

### 2.4. The Role of Biofilm Biology and Microenvironmental Stressors

Biofilms are structured microbial communities attached to surfaces and embedded in an extracellular matrix. Within these communities, gradients of oxygen, nutrients, and pH alter cellular physiology and reduce susceptibility to disinfectants and other stresses [5,8]. Exposure to sublethal disinfectant concentrations or environmental stressors can also induce cross-protection, whereby adaptation to one condition increases tolerance to others. These responses contribute to long-term persistence of pathogens in food-processing environments [42,43].

Consequently, next-generation interventions increasingly rely on biofilm biology. Approaches include surface materials that resist microbial attachment, enzymes that degrade extracellular polymeric substances (EPSs), QSIs that disrupt community coordination, and targeted antimicrobials that eliminate specific strains or resistance elements. Evaluating these strategies on representative surfaces and within complex food matrices remains a critical research priority [40,43,44,45,46].

## 3. Bacteriophages and Phage-Derived Tools

The paradigm shifts outlined above emphasize biological, sequence-level approaches as central elements of next-generation pathogen control. Precision antimicrobials and integrated molecular strategies depend on agents that act on defined targets, and bacteriophages and phage-derived tools represent some of the most mature and practical examples of such targeted interventions in food safety [47,48].

### 3.1. Mechanisms of Action and Host Specificity

Bacteriophages are viruses that infect bacteria and typically follow either a lytic or lysogenic life cycle. In the lytic cycle, a phage attaches to a host cell, injects its genome, redirects host metabolism to produce progeny, and lyses the cell to release new virions. This rapid bactericidal process underlies their application as antimicrobial agents [49,50].

Host recognition is mediated by specific interactions between phage receptor-binding proteins and bacterial surface structures, including proteins, lipopolysaccharides, teichoic acids, or surface polysaccharides. These interactions largely determine host range and explain the narrow specificity of many phages, making receptor characterization critical when selecting phages for food applications [49,51].

Phage performance is strongly influenced by environmental conditions. Temperature, pH, ionic strength, and organic matter affect adsorption efficiency, replication, and particle stability, and must be considered when applying phages in food matrices or on processing surfaces. Formulation strategies, including encapsulation, are commonly used to improve stability under applied conditions [47,52].

Many lytic bacteriophages encode enzymes that promote bacterial lysis and biofilm disruption. Endolysins hydrolyze peptidoglycan to enable cell lysis, while phage-associated depolymerases degrade extracellular polysaccharides and capsule material, facilitating access to biofilm-embedded cells [53]. These enzymes can function independently as antimicrobial proteins or enhance phage penetration into biofilms when used in combination, making them attractive tools for surface sanitation and biofilm control [54,55].

Phage efficacy against biofilms is variable, as biofilm structure can restrict phage diffusion and protect embedded cells, even though planktonic populations are readily reduced [56]. Combining phages with depolymerases, endolysins, or complementary antimicrobials consistently improves biofilm disruption in laboratory and pilot-scale studies. Phage cocktails and phage and enzyme combinations generally outperform single-phage treatments against established biofilms [54,55,57].

Bacterial resistance to phages can arise through modification or loss of phage receptors, restriction modification systems, and CRISPR-based adaptive immunity [58]. These defenses influence phage performance and support mitigation strategies such as phage cocktails, periodic rotation, and engineered phages designed to expand host range or bypass resistance mechanisms [49,59].

Phage specificity is therefore both an advantage and a constraint. Narrow host range limits effects on beneficial microbiota but requires careful matching to target strains or use of multi-phage formulations. Regulatory-approved products illustrate this balance; for example, Listex™ P100 is a lytic phage preparation approved for control of *L. monocytogenes* in foods, demonstrating successful deployment of a well-characterized and targeted phage product [48,52].

### 3.2. Applications in Food Matrices and Processing Environments

Bacteriophages have been evaluated across diverse food types and processing conditions, with multiple studies reporting measurable log-scale reductions in pathogen levels under realistic application scenarios [60]. For *L. monocytogenes*, the lytic phage preparation Listex™ P100 has shown consistent reductions on ready-to-eat (RTE) meats, smoked fish, and fresh produce. Regulatory and risk assessments support its use in specific products, reflecting its efficacy and safety profile [48,61].

Phage treatments applied as sprays, dips, or surface washes typically produce reductions ranging from approximately 1 to 3 log colony-forming units per gram (CFU g^−1^), depending on matrix composition, temperature, phage concentration, and timing of application. For instance, phage cocktails reduced Shiga toxin producing *Escherichia coli* by about 1–2 log CFU g^−1^ on cucumbers and related produce in laboratory studies [62,63].

Meat and poultry processing have been frequent targets for phage application. Field and pilot-scale studies report that phage sprays can reduce *Salmonella* contamination on carcasses, with mean reductions of approximately 1–2 log CFU per surface area. Efficacy depends strongly on temperature, surface characteristics, and organic load, but commercial trials and industry reports indicate practical benefits when phages are integrated into processing workflows [64,65].

Fresh produce presents distinct challenges due to surface complexity and potential internalization. Several studies report initial reductions in *Escherichia coli* (*E. coli*) O157:H7 or *L. monocytogenes* on fresh-cut fruits and leafy greens following phage treatment, although regrowth may occur under favorable storage conditions. Combining phages with additional hurdles, such as refrigeration, washing, or antimicrobial compounds, improves sustained control [61,66].

Phage-derived enzymes, including endolysins and depolymerases, show strong activity against biofilms on food-contact surfaces. Endolysins rapidly lyse Gram-positive bacteria, while depolymerases degrade extracellular matrices that protect biofilm cells [67]. Laboratory and pilot studies show that enzymatic treatments reduce biofilm biomass and increase susceptibility to subsequent sanitation steps. These enzymes are being explored both as standalone interventions and in combination with whole phages [68,69].

Formulation and delivery remain critical determinants of efficacy. Encapsulation, surface immobilization, and incorporation into coatings or films can extend phage stability and activity. Immobilized phages may provide prolonged protection and reduce the frequency of application, provided that immobilization methods preserve infectivity and remain compatible with industrial cleaning practices [70,71].

Phage cocktails are commonly used to broaden host coverage and delay resistance development. Multiple studies and recent reviews show that cocktails often outperform single-phage preparations, particularly in complex matrices that contain diverse strains. Effective cocktail design depends on surveillance data to ensure coverage of relevant food-associated isolates [62,72].

Combined approaches further enhance performance, as phages applied together with mild heat, organic acids, bacteriocins, or nano-enabled formulations often achieve greater pathogen reductions than single interventions [60]. Recent studies indicate that phage–enzyme and phage–antimicrobial combinations improve biofilm disruption and reduce regrowth during storage, reflecting the benefits of complementary mechanisms [69,73].

Despite these advances, limitations remain. Phage efficacy depends on food matrix composition, temperature, and organic load, while biofilms and protected niches can restrict access. In addition, host-range limitations and resistance development require careful phage selection and ongoing monitoring [74]. Regulatory acceptance differs by region, although products such as Listex™ P100 demonstrate a viable route for validated, product-specific applications. Continued field studies, standardized efficacy protocols, and formulation innovations are needed to support broader industrial adoption [48,63,71].

Phage biocontrol in foods is most developed for *L. monocytogenes*. The lytic phage preparation Listex™ P100 has been evaluated by regulatory authorities and received a United States Food and Drug Administration Generally Recognized as Safe notice (GRN 218) for use as an antimicrobial processing aid against *L. monocytogenes* [75,76]. The European Food Safety Authority has also assessed Listex™ P100 for use on ready to eat foods and reported no safety concerns under the approved conditions of application [48].

In addition to activity against planktonic cells, several studies have examined the effect of phage P100 on surface associated populations. Experimental work has shown that *L. monocytogenes* biofilms formed on stainless steel can be reduced or disrupted by phage P100 under controlled conditions [77]. These results indicate that phage treatment can target biofilm associated cells and support its potential use in food processing environments.

For *Salmonella enterica*, phage biocontrol has progressed toward commercial application. A *Salmonella* specific phage cocktail has received a Food and Drug Administration Generally Recognized as Safe notice (GRN 435) for use as an antimicrobial on selected foods. This approval demonstrates a defined regulatory pathway for post-harvest use [78,79].

Phage-based control of Shiga toxin producing *E. coli*, including *E. coli* O157:H7, has also reached regulatory clearance in the United States. EcoShield™, a lytic *E. coli* O157:H7 phage preparation, has been cleared by the Food and Drug Administration as a food contact substance (FCN 1018). Peer reviewed food model studies report that EcoShield™ can significantly reduce *E. coli* O157:H7 contamination on foods. These studies also show that a single application does not necessarily prevent recontamination during later handling or storage [80,81].

Across these pathogens, published studies consistently show that biofilm structure limits access of antimicrobial agents. Phage activity against surface attached communities is often improved by using phage cocktails or by combining phages with enzymes that degrade the biofilm matrix, such as depolymerases, or with lytic enzymes such as endolysins [55,82]. These findings support the use of phage biocontrol not only as a surface decontamination step, but also as a biofilm aware intervention that can be integrated into multi hurdle sanitation programs to address persistent contamination niches [55,77].

**Figure 2:**
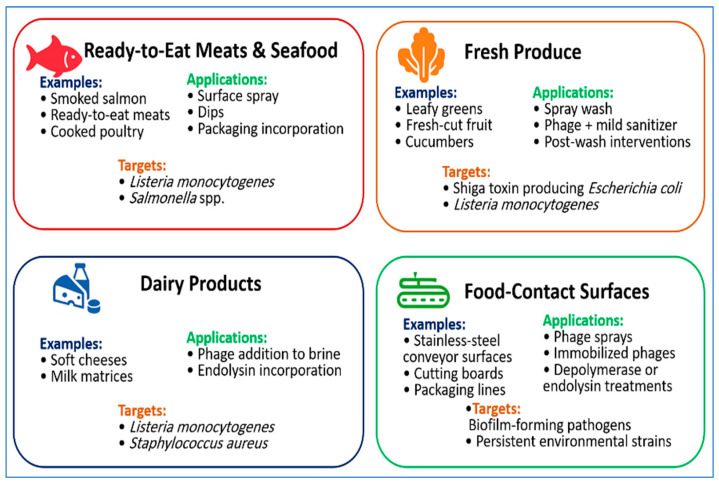
Applications of bacteriophages across food matrices and processing environments. Representative food categories include ready-to-eat meats and seafood, fresh produce, dairy products, and food-contact surfaces. The figure summarizes common application formats, such as surface sprays, dips, wash treatments, packaging incorporation, and immobilized formulations, together with the primary foodborne pathogens targeted in each context. These examples illustrate the practical versatility of bacteriophages and phage-derived enzymes for reducing contamination and supporting food-safety management across different processing settings.

Representative applications of bacteriophages across food matrices and processing environments are illustrated in Figure 2, showing typical food categories, delivery approaches, and target pathogens.

### 3.3. Engineered Phages, Endolysins, and CRISPR-Armed Phages

Bacteriophages have been engineered to enhance killing efficiency, expand host range, and improve biofilm disruption. Modifications include programming phages to deliver biofilm-degrading enzymes or antimicrobial peptides, and several designs show increased activity against biofilm-associated bacteria in laboratory and animal models [83,84]. For example, CRISPR-assisted phage engineering produced variants with improved biofilm targeting and reduced emergence of phage-tolerant *E. coli*, outperforming their wild-type counterparts in vitro and in vivo [83].

Common engineering strategies include deletion of lysogeny-related genes to ensure strictly lytic behavior, modification of receptor-binding proteins to alter host specificity, and insertion of payload genes encoding depolymerases or enzymes that degrade EPS. These modifications enhance biofilm penetration and increase bacterial susceptibility to antimicrobial attack [85,86]. Phage-derived enzymes, particularly endolysins and depolymerases, can also function independently of intact phage particles. Endolysins rapidly cleave peptidoglycan and lyse Gram-positive bacteria when applied externally, whereas depolymerases degrade capsular polysaccharides and EPS, weakening biofilm structure and improving antimicrobial access [86,87].

Experimental studies highlight the applied potential of endolysins. Purified enzymes reduce *Staphylococcus aureus* (*S. aureus*) and *L. monocytogenes* counts in meat and dairy model systems with rapid action and no detectable mammalian cytotoxicity [87,88]. Depolymerase-expressing phages and combined phage–enzyme formulations consistently show greater biofilm removal than phages alone, as matrix degradation increases phage diffusion and biomass reduction [86,89].

CRISPR-armed phages deliver CRISPR–Cas systems into target bacteria, enabling sequence-specific cleavage of essential genes or resistance determinants. This approach can lead to targeted killing or plasmid loss, and proof-of-concept studies demonstrate selective removal of antibiotic resistance genes from mixed populations [83,90]. Delivery remains a key limitation. Phage vectors must efficiently inject CRISPR cargo in complex environments, and the size of CRISPR–Cas systems often exceeds phage packaging limits. Recent solutions include smaller CRISPR effectors and phage-assisted transposases that enable in situ payload insertion, although validation under food-relevant conditions is still required [90,91].

Expanding phage host range through receptor-binding protein modification or tail-fiber exchange may reduce dependence on large phage cocktails. However, broader host specificity raises safety and ecological concerns, necessitating careful host-range evaluation and genomic characterization [85,92]. Manufacturing and formulation also remain practical constraints. Engineered phages and enzymes require scalable production and stable formulations that maintain activity during storage and application. Encapsulation, lyophilization, and surface immobilization have improved stability and controlled release, but each method must be compatible with food matrices and sanitation protocols [93,94].

Safety considerations guide all engineering efforts. Regulatory authorities assess phage products individually, with engineered constructs subject to increased scrutiny relative to natural phages. Risk-assessment frameworks emphasize complete genomic characterization, absence of undesirable genes such as toxins or lysogeny functions, and evaluation of environmental persistence [92,95].

Overall, engineered phages, phage-derived enzymes, and CRISPR-armed systems expand the precision toolkit for controlling foodborne bacteria, with laboratory and preclinical studies demonstrating effectiveness in biofilm control and selective removal of resistance genes [19,67,96,97]. Translational challenges persist, including reliable delivery, formulation stability, host-range management, and regulatory clarity. Addressing these barriers remains essential for advancing engineered phage technologies toward routine food-safety applications [83].

Representative studies evaluating engineered phages, phage-derived enzymes, and CRISPR-enabled constructs across biofilms, food matrices, and in vivo models, together with reported outcomes such as log reductions, biomass removal, and selective strain depletion, are summarized in Table 1.

### 3.4. Regulatory, Safety, and Resistance Concerns

Regulatory acceptance of bacteriophage products for food use is progressing but remains product- and region-specific. In the United States, the lytic phage preparation Listex™ P100 received GRAS status as a processing aid for control of *L. monocytogenes*, with FDA GRAS notices defining intended uses and exposure levels [75]. In Europe, the European Food Safety Authority (EFSA) has evaluated bacteriophages under defined conditions, with approvals dependent on case-by-case assessment and regulatory classification, such as food additive, processing aid, or biocontrol agent. International guidance is emerging to support regulatory harmonization; for example, Organization for Economic Co-operation and Development (OECD) frameworks emphasize product characterization, manufacturing quality, and structured risk assessment to align data requirements across jurisdictions [107].

Available toxicological evidence indicates low direct risk from food-use phage preparations when products are well characterized and free of undesirable genetic elements. Safety evaluations supporting GRAS notices typically report no adverse effects in mammalian models, and regulatory review requires genomic screening to exclude toxin genes, virulence factors, and mobile genetic elements [75]. Phage-derived enzymes, including endolysins, also show favorable safety profiles in experimental studies, with rapid bacteriolysis and low mammalian cytotoxicity. However, each enzyme formulation requires individual assessment, including allergenicity and residual activity testing [87,108]. Worker safety is an additional consideration for spray or aerosol applications. Inhalation exposure, hygiene practices, and sensitization risks must be evaluated, and guidance documents commonly specify application methods to minimize unintended exposure during industrial use [75,107].

Bacterial resistance to phages can occur through receptor modification, restriction–modification systems, and CRISPR–Cas adaptive immunity. These mechanisms are well described and can limit efficacy if phage use is not managed appropriately [49,109]. Mitigation strategies include multi-phage cocktails, rotation of phage preparations, and combination treatments with other antimicrobials, such as enzymes, mild heat, organic acids, or bacteriocins. Experimental and field data indicate that these approaches reduce resistance emergence and improve long-term performance [110,111].

A key ecological concern is phage-mediated horizontal gene transfer, including transduction of antibiotic resistance genes or virulence determinants. Such transfer occurs naturally, but risk increases if preparations contain generalized transducing phages or residual host DNA. Regulatory guidance therefore requires genomic screening to exclude lysogenic or transducing phages and to minimize DNA contamination during manufacturing [95,112]. Recent reviews emphasize the importance of stringent phage selection criteria and robust quality control procedures. While ongoing studies continue to refine risk estimates in food-chain contexts, current consensus supports risk management through rigorous phage selection and manufacturing oversight [95,107].

Commercial deployment of phage products depends on scalable manufacturing using validated host strains, reproducible propagation methods, and purification processes that remove bacterial debris and nucleic acids. Quality control typically includes whole-genome sequencing, sterility testing, and potency assays quantified as plaque-forming units [75,107]. Formulation and storage remain practical challenges because phages are sensitive to temperature, pH, and ultraviolet light. Stabilization methods, such as encapsulation, lyophilization, or incorporation into coatings, can extend shelf life but require validation for activity, release behavior, and compatibility with food matrices and sanitation protocols [20,113].

Engineered and CRISPR-armed phages face increased regulatory scrutiny compared with natural phages. Authorities assess genetic stability, off-target effects, horizontal transfer potential, environmental persistence, and microbiome impacts. Depending on jurisdiction, such products may fall under frameworks for genetically modified organisms or advanced biological agents [85,114]. For food applications, approval will likely require robust data on genetic integrity, absence of mobilizable elements, containment of environmental spread, and environmental risk assessment. Although international harmonization would facilitate development, regulatory pathways remain fragmented [48,107].

Given uncertainties related to long-term ecological effects and resistance dynamics, post-market surveillance is recommended. Monitoring of target populations, resistance development, and environmental distribution supports responsible scaling. Traceability and transparent reporting further enable evidence-based regulation and risk management [95,107].

Regulatory approvals demonstrate a viable pathway for natural, well-characterized phages in food systems. Effective safety management requires selection of strictly lytic phages, exclusion of lysogenic or transducing candidates, comprehensive genomic characterization, and active monitoring for resistance. Engineered and CRISPR-armed phages offer greater precision but face higher regulatory barriers and require expanded safety evaluation. Progress toward routine use will depend on manufacturing standards, surveillance frameworks, and regulatory alignment [48,75,107].

### 3.5. Future Research Directions

Improving phage stability and delivery remains a priority. Encapsulation, lyophilization, and incorporation into coatings extend operational durability, but performance must be validated in commercial food systems. Comparative studies should report standardized metrics, including titer stability, release behavior, and antimicrobial activity in representative matrices under realistic conditions [115,116,117]. Resistance dynamics require measurement under real-world conditions. While laboratory studies document multiple resistance mechanisms, field data are needed to determine frequency, persistence, and fitness costs. Combination strategies, including engineered phages, enzyme cofactors, and mild physicochemical hurdles, consistently reduce regrowth in experimental systems and merit systematic evaluation across food categories [83,115,118,119].

For engineered and CRISPR-armed phages, future work must address genetic stability, containment, and off-target effects prior to food-chain deployment. Regulatory approval will likely depend on comprehensive genomic characterization and environmental risk data [83,120]. Finally, translational research should integrate controlled field trials, harmonized efficacy protocols, and post-deployment monitoring. Documented case studies demonstrating sustained pathogen control, limited resistance emergence, and minimal ecological impact will be essential for broader acceptance by regulators and industry [95,118,121,122].

## 4. Nano-Enabled Antimicrobial Systems

### 4.1. Classes of Nano-Antimicrobials (Organic, Inorganic, Hybrid)

Nano-antimicrobials can be grouped into three practical classes. Inorganic nanoparticles (NPs) include silver (Ag), zinc oxide (ZnO), copper (Cu), and titanium dioxide (TiO_2_), which have been widely studied for use in antimicrobial coatings and additives [123,124]. Organic nano-systems consist of lipid- or polymer-based carriers, such as liposomes, solid lipid NPS, nanostructured lipid carriers, and chitosan or polymeric NPs loaded with EOs or bacteriocins [125,126]. Hybrid systems combine inorganic agents with organic matrices or integrate NPs with enzymatic or peptide antimicrobials to exploit complementary properties [23,127].

Nano-enabled systems often show strong antimicrobial effects in laboratory tests. Their performance can decrease when applied to real foods. Food composition and structure influence how antimicrobial agents interact with microorganisms. As a result, findings from simple test media do not always predict effectiveness in complex food matrices [128]. Representative systems and their documented food-safety applications, together with key regulatory considerations, are summarized in Table 2.

### 4.2. Mechanisms of Nanoscale Antimicrobial Action

NPs exert antimicrobial effects through multiple, often overlapping mechanisms. Metal and metal-oxide NPs can generate reactive oxygen species (ROS), disrupt bacterial membranes, and interfere with nucleic acids and protein synthesis. These mechanisms are well documented for AgNPs and ZnO NPs [123,133]. Some nanomaterials act primarily through contact-mediated activity, in which direct interaction with coated surfaces perturbs bacterial cell envelopes. In contrast, photocatalytic materials, most notably TiO_2_, generate ROS upon light exposure and inactivate microorganisms through oxidative damage [124].

In food-processing environments, organic residues can reduce photocatalytic antimicrobial activity by consuming reactive species and covering active surface sites. The bactericidal effect of TiO_2_ can decrease in wash solutions derived from produce or meat because these solutions contain organic matter such as proteins and phenolic compounds [134]. Photocatalytic performance also depends on the wavelength and intensity of the activating light. Activity can be reduced when organic compounds absorb light or interfere with the photocatalytic reaction [124].

Organic nanocarriers function mainly as delivery systems rather than direct antimicrobials. They enhance solubility of hydrophobic compounds, reduce rapid volatilization of active ingredients such as EOs, and enable controlled release that sustains antimicrobial concentrations at the food or surface interface [125,126]. Hybrid nano-systems integrate these mechanisms by coupling immediate contact activity with prolonged release or by pairing NPs with EPS-degrading enzymes to improve penetration into biofilms and accessibility of embedded cells [135].

Established biofilms are difficult to eliminate. The EPS matrix forms a physical and chemical barrier that limits NP penetration and reduces contact with cells located inside the biofilm [136]. Reviews of NP-based anti-biofilm approaches consistently identify this matrix barrier as a main reason why activity against mature biofilms is lower than activity against planktonic cells [137].

### 4.3. Smart Nanocarriers for Controlled-Release Antimicrobial Delivery

Smart nanocarriers regulate antimicrobial release in response to environmental triggers such as pH, temperature, or enzymatic activity. Lipid-based and polymeric NPs have been developed to stabilize volatile natural antimicrobials, including EOs, and to release them gradually under typical storage conditions, thereby extending antimicrobial effectiveness and shelf life [125,126].

Stimuli-responsive systems, including pH-sensitive polymer matrices and light-responsive coatings, enable on-demand antimicrobial release when contamination or spoilage-related changes occur. Recent reviews emphasize their potential for incorporation into active packaging systems [127,138]. Controlled release behavior can vary across different foods. Moisture level, fat content, and storage temperature influence release rates and reduce predictability. As a result, results obtained from food simulants do not always reflect performance in real food systems [128].

For regulatory and industrial evaluation, controlled-release studies should report antimicrobial release kinetics, retained activity within food matrices, and sustained efficacy over realistic storage durations [139].

### 4.4. Use in Packaging, Surface Coatings, and Wash Treatments

Nano-enabled antimicrobials are applied primarily through active packaging, antimicrobial surface coatings, and wash treatments. In active packaging, nanocarriers or NPs are embedded within polymer films to release antimicrobial agents onto product surfaces or into the packaging headspace. Such systems have demonstrated shelf-life extension in fish, meat, and produce models [127,140].

Antimicrobial nanocoatings applied to stainless steel and polymeric equipment surfaces reduce microbial attachment and biofilm formation under laboratory conditions. Among the most reported approaches are Ag-based coatings and photocatalytic surfaces, which limit surface contamination through contact activity or oxidative mechanisms [135,141].

Long-term performance under industrial sanitation remains a major limitation. Reviews of antimicrobial coatings applied to steel surfaces in food-processing environments emphasize that durability during repeated cleaning, chemical exposure, and mechanical wear is a key barrier to practical use [23]. Clean-in-place procedures also involve strong alkaline and acidic solutions. Cleaning efficiency depends on multiple operational factors, which creates variable exposure conditions that can accelerate surface and coating degradation [142].

Nano-enabled wash treatments use suspensions or emulsions containing nanoencapsulated EOs or metal-oxide dispersions to enhance initial decontamination of fresh produce. Their effectiveness depends on contact time, organic load, and compatibility with subsequent processing steps, highlighting the need for careful integration into existing sanitation workflows [126,127].

Organic load can reduce the effectiveness of antimicrobial interventions used in wash systems. Studies of produce washing show that organic matter in wash water can consume active agents and lower antimicrobial efficacy. This effect can limit the practical impact of wash treatments when wash water is reused or when soil levels are high [143].

### 4.5. Toxicity, Migration, and Regulatory Limitations

Migration of NPs from food-contact materials into food and potential consumer toxicity represent major regulatory concerns. EFSA and other agencies require detailed characterization of particle size, surface chemistry, migration behavior, and toxicological endpoints when evaluating nanomaterials intended for food applications [131,144]. Toxicology studies have reported adverse effects for certain nanomaterials, including TiO_2_ and ZnO, in animal models at high exposure levels. These materials are used in food-related applications such as active and antimicrobial packaging, surface coatings for food-contact materials, and processing aids, underscoring the importance of exposure-based risk assessment that reflects realistic food-use scenarios [145,146].

Migration behavior depends on the polymer matrix, food simulant, temperature, and mechanical stress, making matrix-specific testing essential. Migration studies must therefore follow standardized protocols to generate reliable and comparable data [127,147]. Regulatory approval depends on comprehensive physico-chemical characterization, validated migration testing, and toxicological evidence demonstrating that expected exposure levels are safe. EFSA’s updated guidance on nanotechnology in the food chain outlines technical requirements and evaluation pathways for applicants [144].

### 4.6. Future Applications in Precision Food Safety

Nano-enabled systems can support precision food safety by integrating targeted antimicrobials with sensors and smart packaging technologies. Examples include nanocarriers designed to release antimicrobial agents in response to sensor-detected spoilage markers and packaging films that combine pathogen-binding nanomaterials with downstream signal generation [138,139].

When incorporated into multi-barrier food-safety systems, nanoscale antimicrobials can coordinate with detection technologies and conventional sanitation measures to reduce reliance on broad-spectrum chemical treatments while maintaining microbial control. For wider adoption, nano-enabled antimicrobials must demonstrate consistent performance in complex foods and on industrial surfaces. Future studies should include mature biofilms, realistic soil levels, and processing-relevant conditions such as limited light exposure for photocatalytic systems [124,134,136]. However, broader adoption requires robust evidence of consumer and environmental safety, validated migration and release behavior in representative food systems, and clearer regulatory alignment across jurisdictions [127,144].

Nano-enabled antimicrobials therefore offer flexible tools for pathogen control, ranging from contact-active coatings to stimulus-responsive delivery systems. Their successful deployment depends on balancing antimicrobial performance with demonstrated safety, controlled migration, and regulatory readiness. The following section examines QSIs and anti-virulence strategies, which complement nano-based approaches by disrupting bacterial behavior rather than causing direct lethality.

## 5. QSIs and Anti-Virulence Approaches

### 5.1. Overview of Quorum Sensing in Major Foodborne Pathogens

Quorum sensing (QS) is a cell-to-cell communication system that enables bacteria to coordinate population-level behaviors, including biofilm formation, virulence factor expression, and secretion system activity [148,149]. Signal chemistry and regulatory architecture vary across taxa. Gram-negative bacteria most often use *N*-acyl homoserine lactones (AHLs), whereas Gram-positive bacteria typically rely on peptide autoinducers. Some organisms employ additional or hybrid signaling molecules.

QS regulates adhesion, extracellular matrix production, and toxin expression in several food-relevant pathogens, including *Salmonella enterica*, *L. monocytogenes*, *E. coli*, and *S. aureus* [148,150]. Because QS controls collective behaviors rather than cell viability, it represents an attractive target for strategies aimed at limiting persistence and virulence without exerting strong selective pressure for conventional resistance.

QS regulates biofilm development by coordinating gene expression related to surface attachment, extracellular matrix production, and virulence factor expression [151,152,153]. Disrupting QS signaling interferes with this coordination by preventing signal accumulation or signal recognition, which reduces transcription of QS regulated genes [151,154,155]. As a result, bacterial populations do not synchronize the behaviors needed for stable biofilm formation and coordinated expression of virulence traits [156].

### 5.2. Natural and Synthetic QSIs

A wide range of natural and synthetic compounds exhibit reproducible QSI activity. Ajoene, a sulfur-containing compound derived from garlic, represses QS-regulated gene expression in *Pseudomonas aeruginosa* and enhances antibiotic susceptibility in biofilms. Jakobsen et al. demonstrated QS inhibition by ajoene and reported synergistic enhancement of tobramycin-mediated killing in vitro, together with improved infection clearance in a mouse model [157,158].

Halogenated furanones and synthetic furanone derivatives disrupt AHL receptor interactions and reduce QS-controlled phenotypes. In controlled-delivery formats, furanone-loaded aerogels inhibited *P. aeruginosa* biofilm formation by up to 98.8% and reduced biomass of preformed biofilms in experimental wound-biofilm models [159,160].

Enzymatic quorum quenching offers signal-directed intervention with high specificity. The AHL-lactonase Aii20J, a quorum-quenching enzyme that hydrolyzes *N*-acyl homoserine lactone signaling molecules, reduced early biofilm formation in multispecies in vitro systems by approximately 67.7% at 12 h and 58.1% at 24 h when applied at active concentrations [161]. Immobilized or hybrid enzyme formats, including lactonase-based nanostructures, improve enzyme stability and retain inhibitory activity under harsher environmental conditions. For example, lactonase nanoflowers developed by Chen et al. maintained activity and suppressed pathogen infection in plant–pathogen models [162].

Natural phenolics and EO constituents also show QSI and antibiofilm effects. A eugenol nanoemulsion reduced *L. monocytogenes* biofilm formation on stainless steel by approximately 1.89 log CFU per coupon after 72 h at 25 °C and inactivated mature biofilms to below detection at higher concentrations and short contact times. These findings demonstrate that nanoformulation can enhance both preventive and inactivation performance of QSI-active natural compounds on food-contact surfaces [163].

Synthetic QS antagonists further expand the available chemical space. Tetronamides, methylated denigrins, and related scaffolds produced 60–94% inhibition of *E. coli* biofilm formation in laboratory assays, depending on structure and dose, illustrating the potential of rationally designed QS receptor interference [164].

### 5.3. QS Interference for Biofilm Reduction and Virulence Suppression

QS interference disrupts biofilm development at multiple stages. In early biofilm formation, QS inhibition can reduce adhesion-related behaviors and limit initial surface attachment by blocking the coordinated expression of density-dependent traits [156,165]. During biofilm maturation, disruption of quorum-sensing signaling can decrease EPS production, which weakens biofilm structure and stability [153,166]. QS disruption also reduces the expression of toxins and other virulence factors. This decreases pathogenic potential and can increase susceptibility to antimicrobial treatments [167,168].

Quorum-sensing interference affects multiple stages of biofilm development, including initial attachment, maturation, and virulence factor production, and is commonly associated with reduced biofilm biomass, lower viable cell counts, and increased susceptibility to antimicrobial treatments [156].

Enzymatic quorum quenching using AHL-degrading lactonases consistently produces large reductions in biofilm biomass. In *Pseudomonas* and multispecies systems, lactonases reduced biofilm biomass by 50–77% and enhanced antibiotic efficacy. Aii20J produced inhibition levels of approximately 67.7% at 12 h and 58.1% at 24 h in multispecies in vitro biofilms [169].

Furanone-based QS interference also yields substantial antibiofilm effects. Natural and synthetic furanones reduce AHL-mediated signaling, with delivery-optimized systems achieving near-complete inhibition. Furanone-loaded aerogels suppressed *P. aeruginosa* biofilm formation by up to 98.8%, and treatment of established biofilms resulted in measurable biomass loss [159,160].

Nanoformulated natural QSIs demonstrate both inhibitory and inactivation endpoints. Eugenol nanoemulsion treatment inhibited developing *L. monocytogenes* biofilms at subinhibitory concentrations and rapidly inactivated mature biofilms at higher doses, achieving approximately 7-log CFU reductions in short contact assays [163].

QSIs are most effective when combined with complementary interventions. Ajoene enhanced antibiotic killing of *P. aeruginosa* biofilms, and lactonase treatment increased antimicrobial susceptibility in multidrug-resistant strains. These combined approaches consistently outperform single interventions, supporting their use as adjunct tools in integrated control strategies [157,169].

Representative QS inhibition and quorum-quenching studies reporting quantitative effects on foodborne pathogens and biofilm systems, including biomass reduction, virulence attenuation, and log-scale decreases in viable cells, are summarized in Table 3.

### 5.4. Stability and Scalability Challenges

Despite strong laboratory performance, QSIs and quorum-quenching enzymes face stability limitations in real food environments. Many natural QSIs are chemically unstable, volatile, or prone to adsorption onto food matrices and organic matter, reducing effective concentration at the target site [17,157]. Enzymatic quenchers are sensitive to temperature, pH, and proteolysis, and may lose activity without stabilization.

Formulation strategies such as immobilization, encapsulation, and enzyme NP hybrids improve durability but introduce added cost and processing complexity [162,171,172]. Hybrid lactonase structures and polymer-embedded formulations have demonstrated improved thermal and operational stability, suggesting feasible routes toward durable surface treatments and coatings [162,172,173].

Scalability remains a major constraint. Industrial use requires cost-effective production, standardized potency measures, and application methods compatible with sprays, coatings, or wash steps. High purification costs and regulatory requirements slow translation from laboratory systems to food-processing environments [171,174]. Achieving sufficient local concentrations within heterogeneous biofilms also remains challenging, although controlled-release carriers and combination with physical or enzymatic disruption can improve delivery efficiency [174,175].

### 5.5. Long-Term Potential as Anti-Virulence Interventions

QS interference provides a conceptual advantage by targeting coordinated pathogenic behaviors rather than directly killing cells, thereby reducing selective pressure for classical resistance and limiting effects on beneficial microbiota [176,177]. Nonetheless, resistance to QSIs can still emerge through receptor mutation, signal overproduction, or regulatory rewiring. Modeling and experimental studies highlight the importance of deployment strategies that minimize this risk [178,179].

The most realistic role for QSIs in food safety is as adjunct components of multi-barrier systems. By reducing biofilm formation and virulence, QSIs can sensitize pathogens to sanitizers, enzymes, bacteriophages, or mild physicochemical treatments. Combination approaches also reduce reliance on any single mechanism of control and improve durability [174,180].

For practical adoption, QSI-based strategies must demonstrate stability in target matrices, negligible sensory impact, clear safety profiles, economic feasibility, and post-deployment surveillance for resistance and ecological effects. If these requirements are met, QS interference could become a valuable, lower-pressure tool within integrated food-safety management systems [171,174].

## 6. CRISPR-Based Pathogen Control Technologies

### 6.1. CRISPR-Cas Antimicrobials: Principles and Specificity

CRISPR-Cas antimicrobials rely on programmable, sequence-specific recognition of nucleic acids. A guide RNA directs a Cas effector to a complementary DNA or RNA target, where nuclease activity introduces cleavage that disables the targeted genetic element [20]. Targeting essential chromosomal loci results in lethal genotoxic stress and cell death, whereas targeting plasmids encoding antibiotic resistance or virulence factors can eliminate these elements through plasmid curing without necessarily killing the host cell [20].

This dual functionality underlies both antimicrobial and genetic-sanitization applications. High sequence discrimination, sometimes approaching single-nucleotide resolution, is achievable in principle, but practical specificity depends on guide design, protospacer-adjacent motif constraints of the selected nuclease, and systematic off-target evaluation, which are central features of current CRISPR design pipelines.

### 6.2. CRISPR-Guided Killing of Foodborne Pathogens

Experimental studies demonstrate that CRISPR payloads can selectively eliminate food-relevant pathogens or remove resistance determinants in mixed microbial populations [83]. Engineered bacteriophages carrying CRISPR constructs have achieved targeted depletion of *E. coli* in planktonic cultures, reduction in biofilm-associated cells, and decreased abundance of target strains in animal models, supporting the feasibility of sequence-directed control in complex environments [83].

CRISPR systems designed to target conserved plasmid regions have also successfully eliminated resistance determinants and restored antibiotic susceptibility in heterogeneous communities. These results highlight the complementary roles of lethal targeting and plasmid curing for reducing both pathogen burden and mobile resistance reservoirs [32]. Although most demonstrations remain at laboratory or controlled-model scale, the taxa tested and advances in phage engineering and guide multiplexing support the potential applicability of CRISPR-based control to high-risk foodborne pathogens, including *L. monocytogenes*, *Salmonella*, and pathogenic *E. coli* [83].

CRISPR-based antimicrobials are subject to escape mechanisms that can limit long-term efficacy. Target sequences may mutate or be lost, allowing some bacteria to evade CRISPR targeting, as documented for CRISPR-Cas9 antimicrobials in *E. coli*. These escape mutants evade sequence-specific cleavage and survive despite CRISPR exposure [181]. Bacteria and mobile genetic elements also encode anti-CRISPR proteins that directly inhibit CRISPR-Cas activity by blocking nuclease assembly or target binding, providing functional resistance to CRISPR systems [182,183]. These observations highlight the need for careful guide design, monitoring of target populations, and strategies to reduce the emergence of escape variants in practical applications.

### 6.3. Integration into Sensors and Smart Packaging

Cas effectors with target-activated collateral cleavage activity, particularly Cas12 and Cas13, form the basis of highly sensitive CRISPR-based biosensors. These systems convert sequence recognition into amplified fluorescent, colorimetric, or electrochemical signals and are capable of rapid and low-limit pathogen detection in food matrices [39,184]. When coupled with simple amplification steps, CRISPR diagnostics can deliver results within minutes to hours, enabling point-of-need testing [39,185].

Building on these capabilities, CRISPR sensing elements have been proposed for integration into smart packaging or sensor modules. In such systems, detection of a pathogen could trigger a visible signal or electronic alert, supporting decentralized, real-time monitoring during storage and transport [39,184]. Practical implementation depends on reagent stability, low-cost signal transduction, compatibility with food-contact materials, and validation of shelf life and regulatory safety [46].

### 6.4. Delivery Challenges (Phage, Conjugation, and NPs)

Delivery of CRISPR components into target bacteria remains the primary technical limitation. CRISPR systems are only effective if nucleases and guide RNAs reach the intended host cell in an active form and at sufficient concentration [20]. Bacteriophages are currently the most developed delivery vehicles, as they naturally inject nucleic acids into bacterial cells. CRISPR-armed phages have demonstrated successful payload transfer and target depletion in vitro and in vivo, but their use is constrained by narrow host range, environmental sensitivity, and packaging limits for larger Cas effectors [83,85].

Conjugative plasmids can disseminate CRISPR constructs through microbial communities and provide broader coverage, but their ability to spread horizontally raises concerns related to containment and ecological safety [20]. NP-based carriers, including lipid and polymeric systems, offer an alternative approach by encapsulating Cas ribonucleoproteins and protecting them from degradation while improving penetration into biofilms. Early studies indicate improved delivery efficiency, but questions related to scalability, food-safety toxicology, and regulatory acceptance remain unresolved [186,187].

To address these limitations, hybrid strategies are increasingly favored. Approaches that combine CRISPR-armed phages with depolymerases or stabilizing nanocarriers aim to enhance biofilm access and reduce reliance on a single delivery mechanism [188,189].

Escape and resistance risks can be reduced by combining CRISPR-based approaches with complementary interventions. Guide multiplexing, in which multiple targets are included in CRISPR payloads, lowers the chance that a single mutation can provide escape. Combining CRISPR delivery with bacteriophage cocktails or with lytic enzymes and biofilm-disrupting agents can improve access to target cells and lessen reliance on any single mechanism. Such combinations are important in heterogeneous food-associated microbial communities, where genetic diversity and spatial structure can allow subpopulations to avoid uniform targeting and promote survival [181].

### 6.5. Future Perspectives: Self-Spreading Antimicrobials and Programmable Sanitation

Long-term concepts include controlled self-spreading CRISPR antimicrobials that use conjugative elements or replicating phage vectors to disseminate spacers targeting resistance or virulence genes within microbial reservoirs [96]. While such systems could sustain suppression with fewer applications, their ability to propagate also raises significant concerns regarding containment, reversibility, and unintended ecological effects. Current proposals therefore emphasize layered molecular safeguards and phased environmental testing prior to deployment [20,189].

Another emerging concept is programmable sanitation, in which surfaces, coatings, or packaging incorporate CRISPR delivery modules that remain inactive until a contamination signal triggers localized, sequence-specific intervention. This strategy would shift sanitation from continuous broad-spectrum treatments toward targeted, on-demand control with reduced collateral selection pressure [46,188]. Realization of these approaches requires advances in delivery efficiency, robust molecular containment mechanisms, and clear regulatory and monitoring frameworks to address biosafety and ethical considerations [32,83].

In practical food-safety applications, CRISPR-based tools are unlikely to function as stand-alone interventions. Their most realistic role is within multi-hurdle systems that combine sequence-specific targeting with physical, chemical, or biological controls. Integration with bacteriophages, bacteriocins, QS inhibitors, or engineered surface-active materials can improve robustness and reduce the risk of escape in complex food-processing environments. These layered strategies also help address ecological and regulatory challenges by diffusing selective pressure across multiple control points rather than concentrating it solely on CRISPR targets [181].

**Figure 3:**
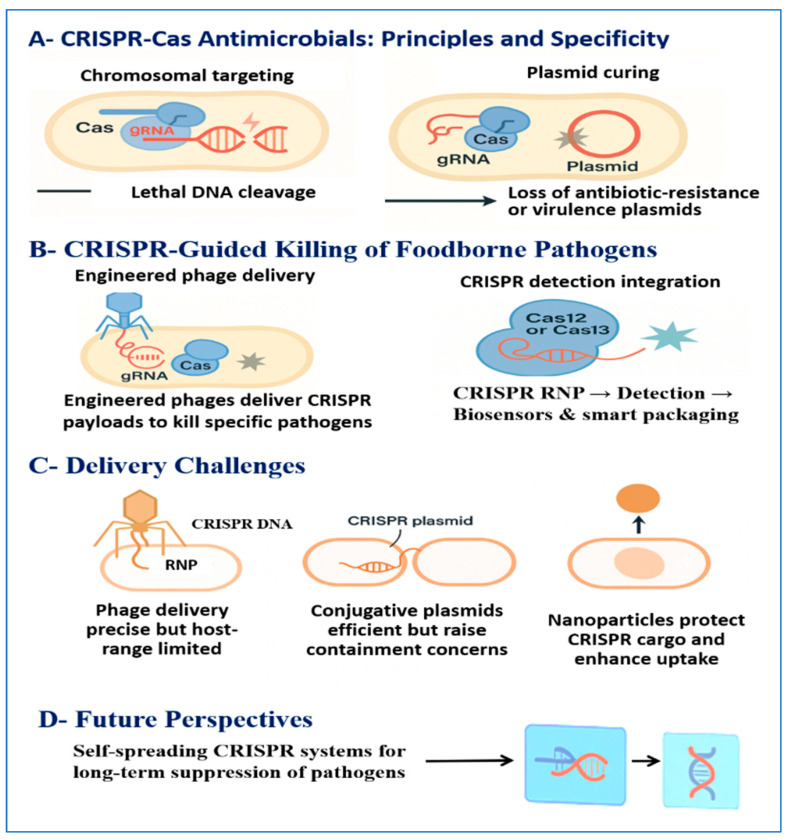
Overview of CRISPR-based foodborne pathogen control technologies relevant to food-safety applications. **Panel A** illustrates sequence-specific CRISPR-Cas antimicrobial mechanisms, including lethal chromosomal targeting and plasmid curing to eliminate antibiotic-resistance or virulence determinants. **Panel B** depicts CRISPR-guided pathogen control through engineered bacteriophage delivery and CRISPR-based detection platforms involving Cas12 or Cas13 for biosensing and smart packaging concepts. **Panel C** summarizes key delivery routes and associated challenges, including phage-based systems, conjugative plasmids, and NP carriers. **Panel D** highlights future perspectives, such as self-spreading CRISPR antimicrobials for sustained suppression of foodborne pathogens and programmable sanitation strategies activated upon detection of contaminant nucleic acids.

The main CRISPR-based pathogen control strategies discussed in this section, including antimicrobial mechanisms, delivery routes, diagnostic integration, and emerging applications, are outlined in Figure 3. Key examples of CRISPR approaches, together with their mechanisms of action, experimental evidence, delivery systems, and relevance to food-safety applications, are summarized in Table 4.

## 7. Natural Bioactive Compounds and Microbial-Derived Alternatives

Natural bioactive compounds and microbial-derived metabolites complement the precision tools discussed in previous sections by aligning with consumer demand for clean-label solutions and by fitting into existing processing workflows with limited infrastructural change [193]. EOs, plant polyphenols, bacteriocins, and postbiotic preparations have demonstrated reproducible activity against major foodborne pathogens and spoilage organisms in model foods and on contact surfaces [21,194,195]. Their application, however, is constrained by instability, sensory impact, and interactions with complex food matrices, highlighting the importance of delivery systems and rational combination with other interventions.

### 7.1. Plant-Derived Compounds (Polyphenols, EOs, and Alkaloids)

Plant-derived antimicrobials include polyphenols, EOs, and alkaloids with diverse chemical structures and mechanisms. These compounds disrupt membranes, interfere with energy metabolism, chelate essential metals, and modulate quorum-sensing pathways in foodborne pathogens [21,195,196]. Polyphenols such as catechins, phenolic acids, tannins, and flavonoids show bacteriostatic or bactericidal activity against *E. coli*, *Salmonella* spp., *L. monocytogenes*, *S. aureus*, and *Clostridium perfringens* in vitro and in model foods [196,197,198]. Susceptibility is compound- and species-specific; for example, gallic acid and catechin derivatives tend to be more active against *S. aureus* and *E. coli*, whereas ellagitannins strongly inhibit *S. aureus* and *C. perfringens* while sparing beneficial *Lactobacillus plantarum* [199,200].

EOs, dominated by terpenoids and phenylpropanoids such as thymol, carvacrol, eugenol, and cinnamaldehyde, exhibit broad antimicrobial activity through membrane disruption, collapse of proton motive force, leakage of cellular contents, and oxidative damage [21,201]. Their efficacy against *L. monocytogenes* and *Salmonella* spp. has been shown in meats, dairy products, fresh produce, and juices, with performance strongly influenced by fat content, water activity, and storage temperature [201,202,203].

Alkaloids including berberine, capsaicin, and piperine display antimicrobial and anti-virulence effects through interactions with DNA, efflux pumps, or cell division proteins, and may act synergistically with antibiotics against *E. coli* and *S. aureus* isolates [196,197]. Their application in foods is limited by bitterness and potential toxicity, favoring controlled-release or surface-restricted use.

Despite strong in vitro activity, food application is limited by binding to proteins or polysaccharides, volatilization or oxidation of EOs, and sensory constraints at effective doses [203,204,205]. These issues motivate encapsulation and structuring strategies described in Section 7.4.

### 7.2. Bacteriocins and Postbiotic Metabolites

Bacteriocins are ribosomally synthesized antimicrobial peptides, largely produced by lactic acid bacteria, that retain activity across pH and temperature ranges relevant to food processing [194,206,207]. Nisin, produced by *Lactococcus lactis*, is the most widely applied bacteriocin and is authorized in many jurisdictions. It disrupts membranes and inhibits cell wall biosynthesis in Gram-positive bacteria, producing rapid inactivation of *L. monocytogenes* and spore-forming *Clostridium* spp. in dairy, meats, and canned products [194,206,208].

Class IIa pediocin-like bacteriocins exhibit strong antilisterial activity and can be applied as purified peptides, fermentates, or via protective cultures. Studies in minimally processed meats and cheeses report 2–4 log reductions of *L. monocytogenes* and delayed spoilage when pediocin-containing cultures are integrated into production systems [194,208,209,210].

Postbiotics, defined by International Scientific Association for Probiotics and Prebiotics (ISAPP) as inanimate microorganisms or their components conferring a health benefit, include organic acids, biosurfactants, exopolysaccharides, and cell wall fragments relevant to food safety [211]. These components lower pH, disrupt membranes, interfere with adhesion, and modulate biofilm formation by pathogens [194,212]. Inactivated lactic acid bacteria preparations retain inhibitory activity against *Salmonella*, *E. coli* O157:H7, and *L. monocytogenes*, offering improved stability, easier standardization, and fewer regulatory concerns compared with live cultures [194,210,211,212].

### 7.3. Synergistic Combinations with Phages, NPs, and QSIs

Natural bioactives rarely achieve sterilization under realistic conditions, driving interest in synergistic combinations with precision tools discussed in Section 3, Section 4 and Section 5. Combined application of bacteriocins and lytic phages often exceeds the efficacy of either agent alone; for example, phage and nisin treatments have achieved greater than 6 log reductions of *L. monocytogenes* in model systems [52,213,214]. Bacteriocins can weaken cell envelopes and reduce emergence of phage resistance, while phage lysis improves access to protected cell populations [213,215,216].

Plant-derived compounds also function as effective adjuvants. EO components such as thymol, carvacrol, and eugenol enhance phage-mediated inactivation of *S. aureus*, *E. coli*, and *Salmonella* on foods when applied at sub-sensory levels [217,218]. Many polyphenols and EO constituents also interfere with QS and early biofilm development, increasing susceptibility to phages, QSIs, and nano-enabled antimicrobials [196,198].

NPs further enhance synergy by acting as carriers or co-treatments. Chitosan and lipid-based NPs improve penetration into biofilms, provide controlled release, and stabilize phages or bacteriocins against environmental stress [203,219,220]. The most effective combinations share mechanistic complementarity, enhanced spatial access to protected niches, and dose reduction that mitigates sensory and safety constraints [217,221,222,223].

### 7.4. Encapsulation and Stability Enhancement Technologies

Encapsulation technologies are central to mitigating volatility and degradation of natural antimicrobials. Liposomes, solid lipid NPs, polymeric capsules, nanoemulsions, and biopolymer-based particles protect EOs, polyphenols, and bacteriocins from oxidation and thermal stress while enabling controlled release [198,219,220,224].

Electrospun nanofibers based on food-grade polymers such as polyvinyl alcohol, zein, or chitosan provide high surface area matrices for gradual diffusion of bioactives and have been proposed for active packaging and edible coatings. Encapsulation efficiencies exceeding 90% and improved dispersibility in aqueous systems have been reported for EO loaded protein and polysaccharide complexes [220,224,225].

Nanoemulsions receive particular attention due to higher antimicrobial efficacy at lower doses compared with coarse emulsions, driven by increased interfacial area and improved contact with microbial cells [219,225]. They have been incorporated into coatings and wash treatments for fresh produce and minimally processed meats with acceptable sensory quality when optimized [205,225,226]. For bacteriocins and postbiotics, alginate, chitosan, and whey protein matrices provide protection from proteolysis and enable gradual release during storage or fermentation [194]. Multifunctional carriers that combine natural bioactives with bacteriocins or phages illustrate how clean-label compounds can be integrated with precision tools in multi-hurdle systems [203,219,225].

### 7.5. Limitations and Processing Challenges

Despite their appeal, natural bioactive and microbial-derived antimicrobials face several interconnected limitations. Sensory impact remains a primary concern, as effective concentrations of essential oils or pigmented plant extracts can alter flavor, aroma, or color beyond consumer acceptance [227,228,229,230].

Matrix interactions further complicate performance; binding to proteins, partitioning into lipid phases, and diffusion barriers in viscous or high-fat foods substantially reduce bioavailability, resulting in large discrepancies between laboratory and real-food efficacy [128,229,231,232,233].

Stability during processing and storage is often limited, as many EOs and phenolics are sensitive to heat, light, oxygen, and pH [27,227,234]. Encapsulation improves resilience but increases formulation complexity and cost [27,228,233]. Additional challenges include compositional variability of natural extracts, leading to reproducibility issues, and diverse regulatory classifications that require region-specific safety and labeling assessments [128,230,232,235].

Cost and scalability also remain limiting factors. Purified compounds and encapsulation technologies add processing steps and expense, which may only be justified in products where clean-label positioning or shelf-life extension provides clear value [232,236]. Consequently, natural bioactives currently show the greatest promise as components of multi-hurdle strategies rather than as stand-alone preservatives. Continued advances in formulation science, delivery control, and regulatory clarity will determine whether these agents transition from niche applications to routine elements of food-safety programs.

## 8. Anti-Biofilm Surface Engineering and Contact-Active Materials

Engineering food-contact surfaces to resist bacterial attachment and disrupt biofilms complements the soluble antimicrobials discussed in earlier sections. Instead of relying on diffusion into foods, these approaches target the earliest states of contamination, i.e., adhesion, colonization, and extracellular matrix development, by tailoring surface topography, chemistry, and responsiveness [237].

### 8.1. Surface Topography, Nano-Texturing, and Anti-Adhesion Strategies

Surface roughness and microdefects strongly influence microbial attachment on food-grade stainless steels. Conventional AISI 304 and 316 steels contain crevices that trap organic residues and facilitate persistent biofilms despite standard cleaning and disinfection protocols [23,238]. Nano-texturing can reverse this tendency by reducing initial microbial attachment and limiting subsequent biofilm development [239]. Electrochemically etched 304 stainless steel coated with Teflon^®^ formed a nanoporous, superhydrophobic surface (contact angle of approximately 151°) that reduced attachment of *E. coli* O157:H7 and *Salmonella typhimurium* by 1.6–2.3 and up to 2.5 log CFU/cm^2^, respectively, relative to untreated steel. Biofilm populations under static and flow conditions were reduced by 2.1–3.0 log CFU/cm^2^, corresponding to greater than 99% reduction in attached cells [240].

Superhydrophobic and slippery designs further limit microorganism to surface contact. Hierarchical fatty-acid crystalline coatings produced from food-grade saturated fatty acids reduced adhesion of *E. coli* and *Listeria innocua*, while slippery liquid-infused porous surfaces prevented more than 95–99% of *P. aeruginosa* and *S. aureus* biofilm accumulation over several days [240,241].

Hydration-layer forming coatings provide an alternative passive strategy. Zwitterionic and highly hydrophilic polymers create dense surface-bound water layers that reduce nonspecific protein adsorption and subsequent bacterial adhesion to near ultra-low-fouling levels. These coatings are particularly relevant where dairy or meat residues form conditioning films that otherwise promote biofilm development [23,242].

### 8.2. Photocatalytic, Catalytic, and Enzyme-Functionalized Surfaces

Photocatalytic oxides such as TiO_2_ and ZnO are widely investigated as contact-active antibiofilm coatings. Upon light exposure, TiO_2_ generates ROS that damage cell envelopes and EPSs. Biofilms of *S. aureus* and *P. putida* grown on TiO_2_-functionalized surfaces showed severe structural disruption after light exposure, with biofilm architecture no longer conferring protection [25,243]. ZnO and TiO_2_ thin films deposited by sol gel or vapor-based techniques have similarly achieved rapid inactivation of *E. coli* while retaining corrosion resistance suitable for food environments [244,245]. In addition, ZnO NPs have been shown to reduce bacterial adhesion and early biofilm formation on stainless steel surfaces and to enhance the efficacy of sodium hypochlorite disinfectants under food-processing conditions [246].

Metallic catalytic coatings, particularly copper alloys, inactivate bacteria through membrane damage and oxidative stress and routinely achieve multi-log reductions within hours. Their use has been proposed for food-processing zones with indirect food contact or regulated exposure [245].

Enzyme-functionalized surfaces provide a more selective approach. Stainless steel surfaces immobilized with lysozyme or lysostaphin retain lytic activity and suppress bacterial growth at the interface. Multilayer and polymer-primed assemblies reduce biofilm formation and facilitate cleaning under flow conditions [242,247]. Bio-inspired nanogel coatings that combine cationic functionality with immobilized enzymes offer durable antibiofilm activity without detectable leaching, provided that enzyme origin and immobilization chemistry meet food-safety requirements [23,242].

### 8.3. Stimuli-Responsive Antimicrobial Surfaces

Stimuli-responsive surfaces remain passive under hygienic conditions but are activated upon contamination-related cues such as pH shifts or microbial metabolites. In packaging contexts, pH-responsive coatings based on alginate, chitosan, or Eudragit^®^ matrices modulate release of essential oils or chlorine dioxide in acidic spoilage microenvironments, extending shelf life of fruits, vegetables, and meats [248].

For equipment surfaces, composite systems combining polymer brushes and antimicrobial enzymes exemplify on-demand activity. A coating integrating poly(acrylic acid) and a bacteriophage-derived endolysin on ZnO nanocolumns created a superhydrophilic, anti-adhesive surface under low contamination. Increased microbial load triggered structural changes that exposed the enzyme, enabling localized contact lysis [248]. Light-activated TiO_2_ coatings similarly provide controllable antimicrobial activation, while responsive polymer brushes can regulate access to embedded antimicrobials in response to environmental conditions [25,245].

### 8.4. Industrial Feasibility and Cleaning-in-Place Compatibility

Industrial adoption requires compatibility with existing hygienic design and cleaning-in-place (CIP) systems. Repeated exposure to hot alkaline detergents, acid treatments, and oxidizing sanitizers can degrade polymeric coatings, alter nano-textures, and reduce adhesion to steel, progressively eroding antibiofilm performance [23].

Reviews consistently identify durability under mechanical abrasion, thermal cycling, and CIP chemicals as the primary barrier to scale-up [23,237]. Although nano-textured and superhydrophobic steels achieve significant reductions in attached cells, wear resistance and cost-effective manufacturing remain unresolved challenges [238,240]. Regulatory acceptance further requires non-migrating materials or compliance with defined migration limits for any releasable components [245].

Rechargeable or removable antimicrobial layers offer a potential compromise, allowing periodic renewal of activity without permanent modification of equipment surfaces. Such approaches may improve long-term feasibility while maintaining cleanability and inspection access [249].

Overall, anti-biofilm surface engineering offers substantial reductions in microbial attachment and biofilm persistence in controlled studies. Translation to food-processing environments depends on sustained performance under CIP conditions, regulatory clearance for food contact, and economical integration with existing stainless-steel infrastructure. Addressing these factors will determine whether contact-active materials become standard components of precision food-safety systems or remain niche interventions.

## 9. Integrating Emerging Technologies into Precision Food Safety Systems

Bacteriophages, nano-enabled antimicrobials, quorum-sensing inhibitors, CRISPR-based tools, natural bioactives, and engineered surfaces are most effective when deployed as coordinated components of a precision food-safety system rather than as stand-alone interventions. Recent advances in multi-hurdle preservation, artificial intelligence (AI) guided monitoring, and digital twin frameworks indicate a shift from generalized control toward targeted interventions matched to specific pathogens, products, and facility niches using real-time data and quantitative risk assessment [38,250,251].

### 9.1. Multi-Hurdle Strategies Combining Next-Generation Tools

The classical hurdle concept has expanded to include biological and molecular interventions such as bacteriophages, bacteriocins, nanoformulations, and natural bioactives. Reviews consistently note that phage efficacy is greatest within multi-hurdle designs, where pairing with organic acids, mild heat, or sanitizers improves log reductions and limits resistance development [252].

Dual biological systems illustrate this principle. Combined bacteriocin and phage treatments extend shelf life and reduce pathogen loads more effectively than either approach alone in dairy, meat, and RTE foods [213,215]. These combinations exploit complementary mechanisms: phages provide strain specificity and amplification, while bacteriocins deliver rapid killing and broaden activity against Gram-positive bacteria.

Natural antimicrobials and nanotechnologies are also incorporated into multi-hurdle surface and packaging strategies. Encapsulated plant compounds combined with physical processes or engineered surfaces can achieve microbial control at lower individual intensities, improving sensory outcomes and reducing chemical load [253,254]. Across systems, three design principles recur: use mechanistically distinct hurdles, optimize their sequence and timing, and validate effectiveness under realistic processing conditions [251,255,256].

### 9.2. AI-Guided Pathogen Prediction and Targeted Interventions

AI and machine learning increasingly convert complex data streams into actionable food-safety decisions. Models integrating sensor outputs, environmental monitoring data, genomics, spectroscopy, and imaging have demonstrated strong performance in contamination detection, spoilage prediction, and traceability analysis [257,258].

Applications relevant to precision intervention include real-time recognition of *Salmonella*, *Listeria*, and *E. coli* contamination using AI-enhanced biosensors and spectral or imaging analysis, often with reported sensitivities above 90% in controlled studies [259,260,261]. Predictive models combining environmental, process, and historical data can forecast high-risk periods or product lots, enabling preemptive intervention rather than reactive recalls [258,262,263]. AI-assisted Hazard Analysis and Critical Control Points (HACCP) systems further support continuous monitoring of time and temperature profiles and hygiene indicators, flagging deviations at critical control points [256,257,264].

Within a precision framework, AI does not replace physical controls; it informs when and where to deploy them. Risk-based outputs can trigger targeted phage or bacteriocin application, adjustment of active-packaging release profiles, or intensified sanitation in zones with elevated predicted risk [257,259].

### 9.3. Digital Twins of Food-Processing Lines

Digital twins, virtual representations of processing systems updated with real-time data, are emerging as tools for optimizing food-safety controls without disrupting production. In food systems, twins have been used to simulate unit operations, optimize process parameters, and improve consistency [38].

Recent applications demonstrate the ability to simulate alternative operating scenarios, integrate predictive microbiology models to estimate pathogen behavior, and optimize cleaning-in-place procedures by visualizing flow patterns and dead zones [38,263,265,266,267]. For precision food safety, the digital twin functions as a systems-level layer that integrates sensor inputs, AI predictions, and intervention options. Conceptually, twins can recommend targeted application of phages, QSIs, or surface-engineered solutions at identified bottlenecks and update predictions dynamically as conditions change. Key challenges remain, including data quality, microbiological validation, and integration costs [38,263].

### 9.4. Pathogen- and Environment-Specific Intervention Mapping

Risk-based food-safety guidance increasingly emphasizes tailoring controls to specific pathogens and environments, particularly for persistent organisms such as *L. monocytogenes* and *Vibrio* spp. [268,269]. Environmental monitoring programs (EMPs) now commonly map facilities into risk zones and align sampling and interventions accordingly [270,271].

Parallel work in spatial and climate-linked risk modeling shows how pathogen risk varies by geography and environment. Examples include climate-driven forecasting of *Vibrio parahaemolyticus* risk and regional vulnerability models linking environmental variables to foodborne disease incidence [272,273,274]. These approaches allow detection technologies and analytics to inform environment-specific decisions rather than generic sanitation practices [275].

Intervention mapping links each pathogen environment scenario to the most appropriate toolset. Persistent *L. monocytogenes* niches may warrant intensified monitoring, targeted biofilm disruption, and anti-adhesive or photocatalytic surfaces in high-risk zones, whereas warm-season *Vibrio* risk in seafood chains may prompt adaptive harvesting controls and biocontrol strategies guided by climate-informed models [268,272,273,275,276]. Digital EMP platforms increasingly support this process by centralizing data and enabling iterative refinement of intervention strategies across time.

## 10. Knowledge Gaps, Regulatory Barriers, and Future Research Priorities

Despite rapid progress, a clear gap remains between experimental success of precision food-safety technologies and their routine use in industrial settings. Many emerging tools perform well under controlled laboratory conditions, but their behavior in complex processing environments is less predictable. Closing this gap requires not only technical refinement, but also closer alignment among research design, regulatory expectations, and industrial practice.

A major limitation is the way microbial systems are studied. Most evaluations focus on single-species cultures, whereas food-processing environments are dominated by mixed microbial communities. Mixed biofilms often display higher persistence and tolerance than monocultures, reducing the reliability of results from simplified models. Future studies must prioritize multispecies systems and test interventions under realistic surface, flow, and soil conditions to improve translational relevance.

Long-term safety is another unresolved issue, particularly for nano-enabled materials. While nano-antimicrobials and coatings offer functional advantages, their stability, migration behavior, and cumulative exposure under repeated processing and cleaning cycles are not well characterized. The absence of long-term exposure and fate data contributes to regulatory caution and delays industrial adoption.

Targeted biological and genetic tools also face practical constraints. Sequence-specific antimicrobials show high precision in laboratory models, but effective delivery in foods and on industrial surfaces remains challenging. Ensuring stability, sufficient local concentration, and containment of unintended effects continues to limit their readiness for routine use. Regulatory pathways for these technologies are still evolving, adding uncertainty for developers and end users.

Biological adaptation further complicates deployment. Bacterial resistance can emerge even against targeted interventions such as bacteriophages, especially with repeated use. Although multi-hurdle and combination strategies reduce this risk, they do not eliminate it, reinforcing the need for surveillance, rotation strategies, and adaptive system design.

Across technologies, a fundamental gap is the lack of harmonized evaluation and validation standards. Studies often employ different foods, conditions, endpoints, and performance metrics, making comparisons difficult and obscuring assessments of industrial readiness. Standardized testing frameworks that link laboratory outcomes to real processing requirements are needed to support regulatory review and investment decisions.

These gaps suggest that future progress will depend less on identifying new control tools and more on optimizing deployment of existing ones. Research priorities should emphasize realistic testing, long-term safety assessment, resistance monitoring, and validation approaches aligned with industrial workflows. Addressing these needs will be essential for translating precision food-safety technologies into reliable, day-to-day components of modern food-production systems.

## 11. Conclusions

Foodborne pathogens remain a persistent challenge to global food safety as AMR increases, biofilms endure, and conventional chemical controls lose effectiveness in complex processing environments. This review shows that emerging precision approaches including bacteriophages, targeted enzymes, gene-guided technologies, natural bioactive compounds, advanced delivery systems, and anti-adhesive surface engineering provide more focused and adaptable ways to control contamination by targeting where and how pathogens persist. The collected evidence supports a shift away from single-solution control toward integrated, multi-hurdle strategies tailored to specific foods, facilities, and risk points. Precision tools are most effective when combined and deployed selectively, rather than applied uniformly across processes. Digital technologies, such as predictive analytics and virtual representations of processing lines, strengthen this framework by linking detection, risk assessment, and targeted intervention in a coordinated manner.

Despite these advances, important barriers remain. Performance under real processing conditions, long-term safety, reliable delivery in complex matrices, resistance management, and regulatory clarity continue to limit broad adoption. Addressing these challenges will require rigorous validation under realistic conditions, harmonized evaluation standards, and sustained collaboration among researchers, industry stakeholders, and regulators. With careful integration of technological innovation, data-driven decision-making, and practical implementation, precision food-safety systems can evolve from promising concepts into dependable tools. Such progress is essential to enhance public health protection while meeting modern industry requirements and consumer expectations for safe and sustainably produced foods.

## Figures and Tables

**Table 1:** Key studies on engineered phages, endolysins, and CRISPR-armed phages.

Key Studies	System/Model	Main Findings	Quantitative Effect
Zhang et al. [98]	Soya milk (4 °C); purified endolysin LysZ5	Rapid lysis of *Listeria* spp. under refrigeration	>4.0 log10 CFU/mL reduction after 3 h at 4 °C
Montañez-Izquierdo et al. [77]	Stainless-steel coupons; 72 h *L. monocytogenes* biofilms; phage P100	Disruption and reduction in established biofilms	3.5–5.4 log10 CFU/cm^2^ reduction (MOI- and time-dependent)
Chibeu et al. [99]	RTE roast beef and cooked turkey; LISTEX™ P100 (10^7^ PFU/cm^2^)	Initial reduction and sustained suppression during refrigerated storage	1.5–2.1 log10 CFU/cm^2^ initially; ~2 log10 lower counts over 28 days at 4 °C
Ibarra-Sánchez et al. [100]	Queso Fresco; endolysin PlyP100 ± nisin	Synergistic control and prevention of pathogen recovery	No detectable cells after 28 days at 4 °C when combined with nisin
Liu et al. [101]	Planktonic cultures and mouse infection models; CRISPR-delivered resistance targeting	Removal of resistance plasmids and restored antibiotic susceptibility	>6-log10 reduction in vitro; ~5-log10 reduction in mice
Lam et al. [102]	Mouse gut model; engineered M13 CRISPR–Cas9 phage	Sequence-specific chromosomal targeting and selective strain depletion	Target strains reduced to 0.0001–0.1% of gut community
Gencay et al. [83]	In vitro biofilms and animal models; engineered CRISPR phages (SNIPR001)	Reduced biofilm activity and limited emergence of tolerant mutants	~45–84% reduction in biofilm activity; dose-dependent CFU reductions in vivo
Zurabov et al. [103]	*Klebsiella pneumoniae* (*K. pneumoniae*) mature biofilms; depolymerase-active phage cocktail	Extensive extracellular matrix disruption and biomass reduction	85–100% biofilm biomass loss; >2–3 log10 CFU reduction
Lu et al. [104]	Duck meat; endolysin LysCP28 (50–100 µg/mL); 4 °C storage	Dose-dependent biofilm removal and pathogen reduction	3.2 log10 CFU/g (100 µg/mL); 3.08 log10 CFU/g (50 µg/mL)
Cha et al. [105]	In vitro biofilms and food-contact surfaces; endolysin LysCSA13	Broad biofilm removal across materials and conditions	~80–90% reduction in biofilm mass
Obeso et al. [106]	Pasteurized milk; recombinant endolysin LysH5	Rapid bactericidal activity with reported synergy	*S. aureus* undetectable after 4 h at 37 °C

**Table 2:** Representative nano-antimicrobial classes, applications, and regulatory considerations.

Nano-Antimicrobial Class	Typical Applications in Food Systems	Key Regulatory/Safety Considerations	References
Photocatalytic TiO_2_NPs	Self-disinfecting food-contact surfaces and equipment; light-activated antimicrobial sanitation	Performance depends on illumination conditions; evaluation focuses on ROS generation, surface stability, and potential particle migration	[129]
Photocatalytic metal-oxide antimicrobial coatings	Antimicrobial coatings for food-contact surfaces and processing lines	Requires nano-specific characterization, controlled activation, and assessment of particle stability during use and cleaning	[130]
Nanomaterials in food and feed (regulatory perspective)	Packaging materials, nano-enabled coatings, and applications requiring pre-market approval	Emphasizes particle size and surface characterization, migration testing, toxicology, and exposure modeling	[131]
NPs in the food chain: regulatory and toxicological review	Packaging, coatings, and processing aids evaluated by food-safety authorities	Highlights exposure estimation, compliance with Europe nano-specific guidance, and integration of toxicological and migration data	[132]

**Table 3:** Verified QSI and quorum-quenching studies with quantitative outcomes.

Pathogen/Model System	Treatment	Experimental Matrix	Verified Quantitative Outcome	References
*P. aeruginosa* PAO1 (in vitro; murine model)	Ajoene (garlic-derived QSI)	In vitro biofilms; mouse infection model	QS gene suppression; enhanced tobramycin-mediated biofilm killing; improved infection clearance	[157]
*P. aeruginosa*	Halogenated furanone in polyvinyl alcohol aerogels	In vitro biofilm assays	Up to 98.8% inhibition of biofilm formation; reduced biomass in pre-formed biofilms	[159]
Multispecies oral/subgingival biofilms	Aii20J AHL-lactonase	In vitro multispecies biofilm models	Biomass reduction of ~30–60% across tested communities	[161]
*P. aeruginosa*	Nano-hybrid quorum-quenching enzyme combined with antibiotic	Planktonic and biofilm assays	~97% attenuation of QS-regulated virulence factors	[170]
*L. monocytogenes*	Eugenol nanoemulsion	Stainless-steel coupons	1.89 log CFU/coupon reduction in developing biofilms; ~7 log CFU inactivation at higher concentrations	[163]
*E. coli*	Synthetic tetronamide and denigrin analogues	In vitro biofilm assays	60–94% biofilm inhibition depending on compound and dose	[164]
Mixed/pathogen model systems	AHL-lactonase hybrid nanoflowers	In vitro and plant–pathogen assays	Strong QS suppression with significant biofilm reduction	[162]

**Table 4:** Summary of CRISPR-Based Pathogen Control Technologies and Supporting Evidence.

Subsection	Focus/Mechanism	Key Supporting Evidence	References
CRISPR-Cas antimicrobials: principles and specificity	Guide RNA–directed Cas nucleases target chromosomal or plasmid DNA with sequence-level specificity	Chromosomal cleavage induces lethal genotoxic stress; plasmid targeting results in curing. Specificity depends on guide design, protospacer adjacent motif constraints, and off-target screening	[19]
CRISPR-guided killing of foodborne pathogens	Selective elimination of targeted bacterial strains or resistance determinants	Phage-delivered CRISPR systems reduce target bacteria in cultures, biofilms, and animal models; plasmid targeting restores antibiotic susceptibility	[19,83]
Integration into biosensors and smart packaging	Collateral cleavage activity of Cas12 and Cas13 enables rapid nucleic acid detection	SHERLOCK and DETECTR platforms support sensitive pathogen detection and proposed smart packaging applications	[190,191]
Delivery challenges (phage, conjugation, and NPs)	Transport of CRISPR components into target bacteria	Phage delivery validated in vitro and in vivo but constrained by host range; conjugative plasmids raise ecological concerns; and NP carriers improve delivery in model systems	[19,83,90,192]
Future perspectives: self-spreading antimicrobials and programmable sanitation	Replicative CRISPR dissemination and detection-triggered sanitation concepts	CRISPR–transposase and replicating phage systems demonstrate controlled propagation; studies emphasize containment and regulatory oversight	[19,90,190]

## Data Availability

No new data were created or analyzed in this study.
